# Complete mitochondrial genome sequence of *Macromia daimoji* Okumura, 1949 (Odonata: Macromiidae)

**DOI:** 10.1080/23802359.2018.1450683

**Published:** 2018-03-14

**Authors:** Min Jee Kim, Su Yeon Jeong, Ah Rha Wang, Junghwa An, Iksoo Kim

**Affiliations:** a Department of Applied Biology, College of Agriculture and Life Sciences, Chonnam National University, Gwangju, Republic of Korea;; b Animal Resources Division, National Institute of Biological Resources, Incheon, Republic of Korea

**Keywords:** Mitochondrial genome, Odonata, *Macromia daimoji*, Macromiidae, Endangered species

## Abstract

The dragonfly *Macromia daimoji* Okumura, 1949 (Odonata: Macromiidae) has been listed as an Endangered insect in South Korea. We sequenced the complete 15,198 bp mitochondrial genome (mitogenome) of this organism, which is the first mitogenome sequence reported from the family Macromiidae. The genome includes a typical set of genes [13 protein-coding genes (PCGs), 2 rRNA genes, and 22 tRNA genes) and one non-coding region with an arrangement identical to that observed in most insect genomes. Phylogenetic analyses using concatenated sequences of the 13 PCGs and 2 rRNA genes using the Bayesian inference (BI) method placed Macromiidae, represented by *M. daimoji*, as a sister group to Libellulidae with the highest nodal support [Bayesian posterior probabilities (BPP) = 1]. Unlike conventional phylogenetic analysis, the suborders Anisozygoptera and Zygoptera formed a strong sister group (BPP =1), justifying the use of different molecular markers for phylogenetic analysis.

*Macromia daimoji* Okumura, 1949 (Odonata: Macromiidae), which is listed as an Endangered species in South Korea, is distributed in mid-Northern South Korea, Japan, and Southern Russia (Jeong 2012; http://www.me.go.kr/home/web/main.do). In Korea, limited ecological information for this species is available (Jeong 2012).

An adult male *M. daimoji* was collected from Yeongcheon gun, Gwangwon-do Province (38° 5' 47.20'' N, 127° 4' 29.40'' E), South Korea in 2009. This voucher specimen was deposited at the Chonnam National University, Gwangju, Korea, under the accession no. CNU7046. Using DNA extracted from the hind legs, four long overlapping fragments (LFs; *COI*-*ND5*, *ND5*-*CytB*, *CytB*-*srRNA*, and *srRNA*-*COI*) were amplified using four sets of primers designed from the available mitogenomes of Odonata (Lee et al. 2009; Wang et al. 2015; Yu et al. 2016; Jeong et al. 2017). Subsequently, these LFs were used as templates for amplifying 24 short fragments. The sequence data has been deposited in GenBank under the accession number MF990748.

We performed phylogenetic analysis using the concatenated nucleotide sequences of 13 protein-coding genes (PCGs) and 2 rRNA genes of 24 mitogenome sequences from Odonata. An optimal partitioning scheme (6 partitions) and substitution model (GTR + Gamma + I) were determined using PartitionFinder 2 and the Greedy algorithm (Lanfear et al. 2012; Lanfear, Calcott, Kainer, et al. 2014; Lanfear, Frandsen, et al. 2016). Bayesian inference (BI) analysis was conducted using Mr. Bayes ver. 3.2.2 (Ronquist et al. 2012) implemented on the CIPRES Portal ver. 3.1 (Miller et al. 2010).

The complete 15,198 bp mitogenome of *M. daimoji* was composed of 2 rRNAs, 22 tRNAs, 13 PCGs, and 1 major non-coding region referred to as the A + T-rich region (467 bp). The arrangement of this genome was identical to that typically observed for other insects (Cameron 2014). The A/T content of the whole mitogenome was 73.5%; however, it varied among the genes as follows: 86.4%, A + T-rich region; 75.5%, lrRNAs; 75.2%, srRNAs; 74.2%, tRNAs; and 72.4%, PCGs. Twelve PCGs had the typical ATN start codon, whereas *ND1* had the atypical TTG codon. Ten of the 13 PCGs had a complete stop codon; however, *COI*, *COII*, and *ND5* had an incomplete stop codon, T.

Results of phylogenetic analysis using the BI algorithm indicated sister relationship between Libellulidae and the newly added family, Macromiidae, with a strong nodal support (Figure 1; BPP = 1), as has been previously shown using *COI*, 16S rRNA, 28S rRNA, and EF1-α sequences (Kim et al. 2014). Zygoptera was monophyletic with the highest nodal support (BPP = 1), and all the zygopteran superfamilies and families represented by more than one species were consistently and strongly supported as monophyletic groups. On the other hand, monophyletic Anisoptera was only moderately supported (BPP = 0.71), whereas the sister relationship between Aeshnoidea and Libelluloidea was also strongly supported (BPP = 1). The sister relationship between Zygoptera and Anisozygoptera (BPP = 1) was unconventional (Rehn 2003; Davis et al. 2011; Kim et al. 2014), but recent mitogenome-based phylogenetic results consistently supported the sister relationship between these two suborders (Yong et al. 2016; Jeong et al. 2017). We believe that in future, more species representing diverse taxonomic groups will help in understanding the odonate phylogeny.

**Figure 1.**
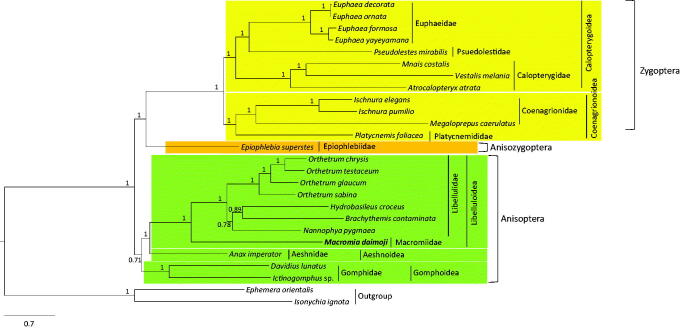
Bayesian inference (BI) method-based phylogenetic tree for order Odonata obtained using concatenated sequences of 13 PCGs and 2 rRNAs. The numbers at each node indicate Bayesian posterior probabilities (BPP). The scale bar indicates the number of substitutions per site. Two species belonging to order Ephemeroptera were used as outgroups. GenBank accession numbers are as follows: *B. contaminata*, KM658172 (Yu et al. 2016); *H. croceus*, KM244659 (Tang et al. 2014); *N. pygmaea*, KY402222 (Jeong et al. 2017); *O. chrysis*, KU361233 (Yong et al. 2016); *O. glaucum*, KU361232 (Yong et al. 2016); *O. Sabina*, KU361234 (Yong et al. 2016); *O. testaceum*, KU361235 (Yong et al. 2016); *Ictinogomphus* sp., KM244673 (Tang et al. 2014); *D. lunatus*, EU591677 (Lee et al. 2009); *A. imperator*, KX161841 (Herzog et al. 2016); *P. mirabilis*, FJ606784 (unpublished); *E. formosa*, HM126547 (Lin et al. 2010); *E. ornata*, KF718295 (unpublished); *E. decorata*, KF718294 (unpublished); *E. yayeyamana*, KF718293 (unpublished); *V. melania*, JX050224 (Chen et al. 2015); *A. atrata*, KP233805 (unpublished); *M. costalis*, KU871065 (Lorenzo-Carballa et al. 2016); *I. pumilio*, KC878732 (Lorenzo-Carballa et al. 2014); *I. elegans*, KU958378 (Feindt et al. 2016a); *P. foliacea*, KP233804 (unpublished); *M. caerulatus*, KU958377 (Feindt et al. 2016b); *E. superstes*, JX050223 (Wang et al. 2015); *E. orientalis*, EU591678 (Lee et al. 2009); and *I. ignota*, HM143892 (unpublished).
